# Genomic characterization of drug-resistant *Mycobacterium tuberculosis* complex in western Kenya reveals predominance of isoniazid resistance and discordance with routine susceptibility testing

**DOI:** 10.3389/ftubr.2026.1855780

**Published:** 2026-06-11

**Authors:** Susan Musau, Susan Odera, Victor Moses Musyoki, Eleanor I. Lamont, Noel Onyango, Bob Morrison, Wilfred Murithi, Steve Wandiga, Videlis Nduba, David Sherman, Marianne W. Mureithi

**Affiliations:** 1 Department of Medical Microbiology and Immunology, University of Nairobi, Nairobi, Kenya; 2 Tuberculosis and HIV Co-Infection Training Program, University of Nairobi, Nairobi, Kenya; 3 KAVI-Institute of Clinical Research, University of Nairobi, Nairobi, Kenya; 4 Department of Microbiology, University of Washington, Seattle, WA, United States; 5 Centre for Respiratory Diseases research, Kenya Medical Research Institute, Nairobi, Kenya; 6 Center for Global Health Research, Kenya Medical Research Institute, Kisumu, Kenya

**Keywords:** diagnostic, drug-resistant tuberculosis, genomic surveillance, Kenya, *Mycobacterium tuberculosis* complex, whole-genome sequencing

## Abstract

**Introduction:**

Whole-genome sequencing (WGS) data on resistance patterns in western Kenya remain limited. We evaluated resistant patterns of *Mycobacterium tuberculosis* complex (MTBC) isolates from western Kenya based on routine line probe assay (LPA) and/or phenotypic drug-susceptibility testing (DST) using targeted WGS.

**Methods:**

In this cross-sectional study, 1,053 archived MTBC isolates from 12 counties in western Kenya were analyzed. An enriched subset of 316 isolates was selected for WGS, of which 290 produced high-quality genomes. Genomic data were analyzed to determine lineage distribution and identify drug resistance mutations using WHO mutation catalogue.

**Results:**

Out of the 1,053 isolates, 127 (12%) showed resistance to at least one anti-TB drug. Isoniazid resistant-rifampicin susceptible (Hr-TB) isolates were the most common (52; 40%), followed by rifampicin-resistant (33;26%) and multidrug-resistant (23;18%). The predominance of Hr-TB is consistent with reports from Kenya and other high-burden settings. Enriched WGS subset identified resistance-associated mutations in 43/290 (14%) isolates, with similar resistance patterns. Rifampicin resistance was mainly associated with *rpoB* (S450L), while isoniazid resistance was dominated by *katG* S315 T and *inhA* promoter mutations. Resistant isolates were mainly concentrated in Kisii, Migori, Homa Bay, and Siaya, predominantly within Lineages 4 and 3. Discordance was observed between routine testing and WGS (*p* = 0.026); approximately 86% of discordant isolates carrying mutations of uncertain significance.

**Conclusion:**

Western Kenya shows geographic DR-TB heterogeneity with predominant Hr-TB, which rifampicin-based diagnostics risk under-detection. Despite observed discordance, Integrating WGS with routine testing will strengthen surveillance, improve isoniazid resistance detection, and support appropriate treatment management in high-burden settings.

## 1 Introduction

Tuberculosis remains a critical global challenge, with 10.8 million cases and 1.25 million deaths in 2023. Drug-resistant strains (MDR/RR-TB and XDR-TB) present significant barriers to elimination, showing lower treatment success rates and contributing to nearly 25% of TB mortality (1–4). Resistance primarily arises from spontaneous mutations in genes such as *rpoB*, *katG*, and *inhA* for first-line drugs, and *gyrA/B* and *rrs* for second-line agents (5–9). Validating the reliability of these markers is essential for optimizing treatment algorithms in high-burden settings.

In Kenya, surveillance relies heavily on conventional genotyping like Spoligotyping and mycobacterium interspersed repetitive unit – variable-number tandem repeats (MIRU-VNTR), which interrogates only ∼1% of the genome, creating gaps in detecting non-canonical mutations or resistance to newer drugs (10–15). This is especially problematic in western Kenya, a high-burden region with significant HIV co-infection where comprehensive whole-genome sequencing (WGS) data is scarce (16–20). Implementing localized WGS is essential to resolve the genetic architecture of local strains and inform data-driven treatment and public health strategies.

WGS has emerged as a powerful tool for TB drug-resistance surveillance, providing genome-wide coverage (∼90%) and enabling simultaneous detection of resistance-associated mutations across both first- and second-line anti-tuberculosis drugs (12). WGS facilitates high-resolution identification of single-nucleotide polymorphisms and structural variants, improves discrimination between closely related strains, and supports prediction of drug-resistance phenotypes (21–23). Studies from high-burden settings, including East Africa, have demonstrated the utility of WGS in predicting drug resistance, guiding treatment decisions, and improving patient outcomes (24–28).

Given the growing burden of drug-resistant TB and the limited availability of comprehensive genomic data in Western Kenya, there is a critical need for WGS-based drug-resistance profiling of MTBC isolates from this region. We hypothesize that WGS can provide high-resolution detection of both canonical and non-canonical resistance mutations, offering superior resistance profiling compared to routine diagnostic assays (29).This study aimed to characterize drug-resistance mutations in MTBC isolates from Western Kenya and evaluate concordance between WGS and routine diagnostics.

## 2 Methods

### 2.1 Study design and setting

This retrospective, cross-sectional laboratory study characterized genomic mutations associated with drug-resistant *M. tuberculosis complex* in western Kenya. Archived isolates were sourced between January 2021 and December 2022 from the KEMRI/CGHR Tuberculosis Laboratory, a regional reference center serving 12 counties: Kisumu, Migori, Siaya, Homa Bay, Kisii, Nyamira, Busia, Kakamega, Vihiga, Bungoma, Bomet, and Kericho.

The study included culture-positive isolates obtained from patients of all age groups (pediatric and adult populations) presenting with presumptive or confirmed drug-resistant tuberculosis complex**.** Eligible cases were identified in accordance with the Kenya National TB Diagnostic Algorithm (30). This included Xpert MTBC positive cases (with or without rifampicin resistance), Individuals at high risk for drug-resistant TB (such as treatment failures and relapses), special risk populations (such as symptomatic healthcare workers, residents of correctional facilities, and individuals in refugee camps), follow-up DR-TB patients and selected new smear-positive/Xpert-negative cases, for MTBC and nontuberculous mycobacteria (NTM) differentiation. Isolates negative on Mycobacteria Growth Indicator Tube (MGIT) culture or contaminated were excluded.

Prior to archiving and selection for sequencing, all clinical specimens underwent routine diagnostic evaluation according to the national diagnostic algorithm. This workflow included primary processing via smear microscopy (fluorescence microscopy for acid-fast bacilli), followed by liquid culture using the MGIT 960 system. Isolates with positive growth confirmation were further evaluated using Line Probe Assays (LPA), specifically targeting mutations associated with rifampicin and isoniazid resistance and/or phenotypic Drug Susceptibility Testing (DST) using the critical concentration method in liquid media to confirm resistance profiles alongside the molecular results.

**Figure 1:**
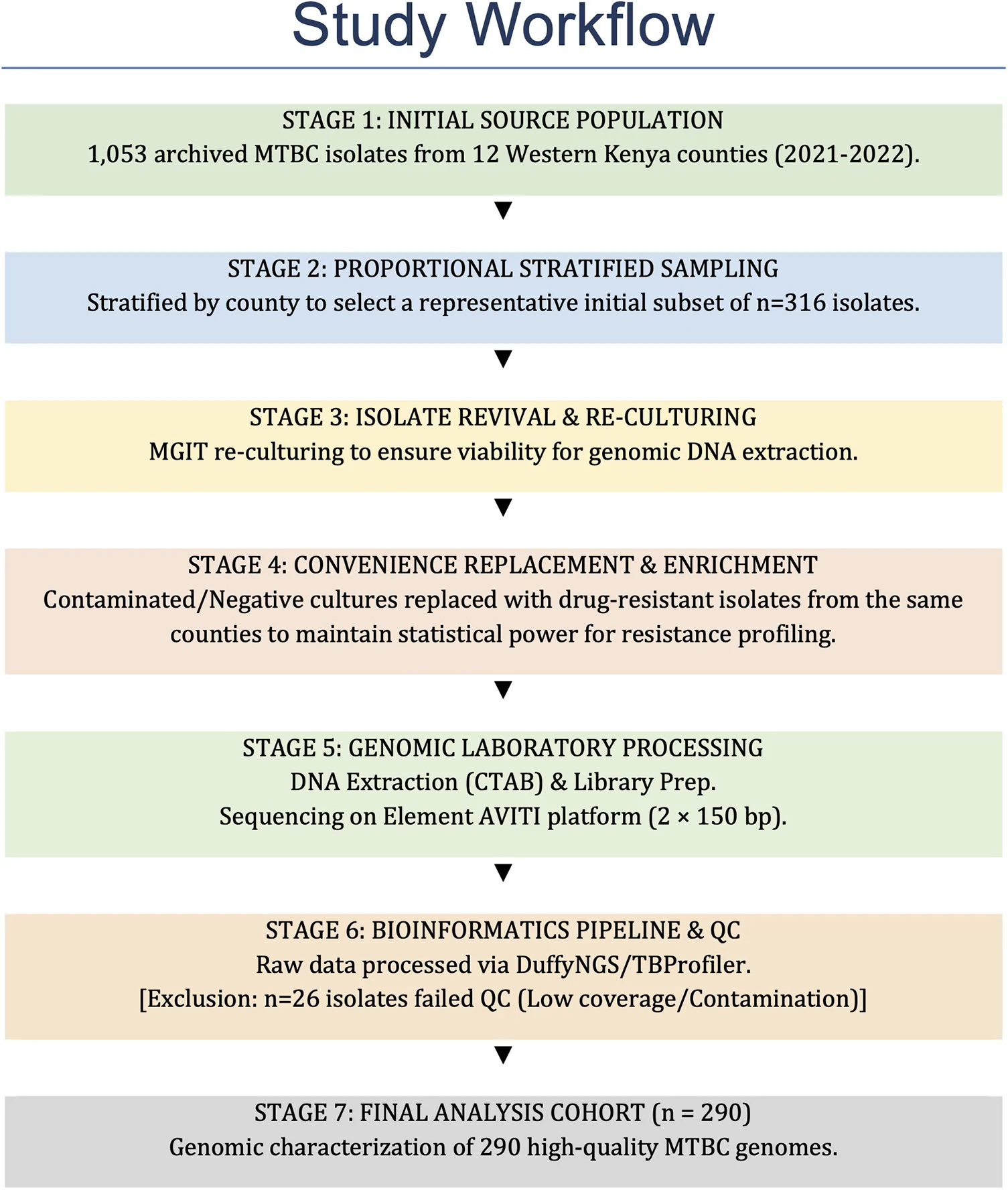
Study workflow and isolate selection for genomic characterization. MTBC, *Mycobacterium tuberculosis* complex; WGS, whole-genome sequencing; DST, drug-susceptibility testing; LPA, line probe assay; QC, quality control. Selection strategy transitioned from proportional stratified sampling to convenience enrichment to prioritize drug-resistance characterization.

Of the 1,053 isolates identified, 316 were selected for sequencing, with 290 producing high-quality WGS data for the final analysis. Due to the retrospective nature of the study, a convenience sampling method was used to replace non-viable isolates with archived drug-resistant strains from the same respective counties as elaborated in Figure 1. This targeted enrichment ensures statistical power for resistance profiling but is not intended to reflect true population prevalence. The comprehensive distribution of both the total collection (*N* = 1,053) and the sequenced subset (*n* = 290) across the 12 counties and two-year timeframe is detailed in Supplementary Table 1.

Of the 290 *Mycobacterium tuberculosis* complex (MTBC) isolates included in this study, routine diagnostic data were available in varying proportions due to the retrospective nature of the archived samples. Specifically, 154 isolates (53.1%) had only Line Probe Assay (LPA) results available, 15 isolates (5.2%) had only phenotypic Drug Susceptibility Testing (pDST) via BACTEC MGIT, and 121 isolates (41.7%) had results for both LPA and pDST. Whole-genome sequencing (WGS) was performed on all 290 isolates to serve as the definitive benchmark for drug resistance characterization.

### 2.2 Socio-demographic data collection

Socio-demographic information (age, sex, and county of origin) was retrieved from laboratory records and linked to isolate identification numbers. All data were anonymized before analysis.

### 2.3 Laboratory procedures

#### 2.3.1 Sample processing and culture

Stored isolates were decontaminated using the N-acetyl-L-cysteine (NALC)-NaOH method and cultured in MGIT liquid medium supplemented with Oleic Acid-Albumin-Dextrose-Catalase (OADC) and Polymyxin B, Amphotericin B, Nalidixic acid, Trimethoprim, and Azlocillin (PANTA). Growth was monitored via the BACTEC™ MGIT™ 960 system, with purity verified on blood agar and species identification confirmed using the BD MGIT™ TBc test. Drug-susceptibility testing (DST) covered first-line (H, R, E, Z) and second-line drugs (amikacin, kanamycin, capreomycin, and fluoroquinolones). Resistance was determined phenotypically via the MGIT 960 SIRE/PZA system and genotypically using GenoType® MTBDRplus and MTBDRsl line probe assays. These results served as the reference standard for validating WGS-based resistance predictions.

#### 2.3.2 Genomic DNA extraction

High-quality genomic DNA was extracted using a modified cetyltrimethylammonium bromide (CTAB) method optimized for mycobacterial lysis and purity (31).

#### 2.3.3 Library preparation and whole genome sequencing

Genomic libraries were prepared using the NEBNext Ultra II FS DNA Library Preparation Kit (New England Biolabs, USA) following the manufacturer's protocol. Fragment size distribution was confirmed using an Agilent Bioanalyzer, and library concentration was quantified using the KAPA Library Quantification Kit (Roche, USA). Libraries were normalized, pooled, and sequenced on the Element AVITI platform (Element Biosciences, USA) to generate paired end reads (2 × 150 bp). Resulting FASTQ files were processed for downstream bioinformatics analyses.

### 2.4 Bioinformatics analysis

Raw sequencing data from the Element AVITI platform was processed using the DuffyNGS pipeline (v1.8.0; https://github.com/robertdouglasmorrison), where the QuickQC module facilitated quality assessment, adapter trimming, and the removal of low-quality bases. High-fidelity reads were aligned to the *M. tuberculosis* H37Rv reference genome using Bowtie2 (32), with alignment metrics summarized via Samtools (33). Variant detection, including SNPs and indels, was performed using Samtools/BCFtools (34) algorithms integrated within the DuffyNGS VariantCalls module. In this workflow, the DuffyNGS pipeline provided the primary bioinformatics framework for raw data pre-processing, alignment, and high-resolution variant calling.

Genotypic drug resistance was characterized using TBProfiler (v4.3.0) to annotate variants against a curated database of resistance determinants. The analysis focused on key loci for first-line agents (e.g., *rpoB, katG, inhA, pncA*) and an extensive panel of genes associated with second-line and newer drugs, such as fluoroquinolones, bedaquiline, and linezolid (30). Resistance was interpreted according to the WHO mutation catalogue v2 (35), specifically including mutations classified as “associated with resistance” or “associated with resistance – interim.”

### 2.5 Statistical analysis and data visualization

The agreement between WGS profiles and phenotypic drug susceptibility testing (DST) and/or Line Probe Assay (LPA) results was statistically validated using McNemar's test with continuity correction. Due to the retrospective and anonymized nature of the laboratory-sourced isolates, statistical analysis was limited to describing the frequency of resistance mutations across lineages and geographic regions to define the molecular landscape of drug-resistant TB in western Kenya.

## 3 Ethical considerations

This study used de-identified archived isolates and corresponding laboratory data with no direct patient contact. Ethical approval was obtained from the Kenyatta National Hospital–University of Nairobi Ethics and Research Committee (KNH-UoN ERC) and the Kenya Medical Research Institute Scientific and Ethics Review Unit (SERU). Additional authorization was granted by the National Commission for Science, Technology and Innovation (NACOSTI), the KEMRI-CGHR Director, and the National TB Programme.

## 4 Results

### 4.1 Drug resistance profiles as determined by routine diagnostics in western Kenya

Of the 1,053 isolates, 926 (87.9%) were classified as drug-susceptible, while 127 isolates (12.1%) demonstrated resistance to at least one anti-tuberculosis drug. Among the resistant isolates, isoniazid-resistant tuberculosis (Hr-TB), defined as resistance to isoniazid with susceptibility to rifampicin, was the most frequently identified resistance pattern, accounting for 52 isolates (4.9% of all isolates; 40.9% of resistant isolates).

Rifampicin-resistant tuberculosis (RR-TB) was detected in 33 isolates (3.1%), while multidrug-resistant tuberculosis (MDR-TB), defined as resistance to both rifampicin and isoniazid; was identified in 23 isolates (2.2%). In addition, two isolates (0.2%) were classified as pre-extensively drug-resistant tuberculosis (pre-XDR-TB), indicating MDR-TB with additional resistance to fluoroquinolones.

Resistance to second-line drugs was also observed in a small number of isolates. Capreomycin resistance (CAP-R) was detected in five (0.5%) isolates, while fluoroquinolone resistance (FQ-R) not associated with MDR/RR-TB was identified in one (0.1%) isolate. Additionally, 11 (1%) isolates exhibited resistance to other first-line drugs, including ethionamide (ETH) and pyrazinamide (PZA) (Table 1).

**Table 1:** Distribution of drug resistance and susceptibility among *MTBC* isolates in western Kenya as determined by routine diagnostic methods (*n* = 1,053).

Classification	*n* = 1,053 (%)
Drug-susceptible	926 (87.9)
Hr-TB	52 (4.9)
RR-TB	33 (3.1)
MDR-TB	23 (2.2)
pre-XDR-TB	2 (0.2)
CAP-R	5 (0.5)
FQ-R	1 (0.1)
Other first-line resistance (ETH/PZA)	11 (1.0)
	1,053 (100)

Hr-TB, resistance to isoniazid with susceptibility to rifampicin; RR-TB, rifampicin-resistant tuberculosis; MDR-TB, resistance to both rifampicin and isoniazid; pre-XDR-TB, indicating MDR-TB with additional resistance to fluoroquinolones; CAP-R, capreomycin-resistant; ETH, ethionamide; PZA, pyrazinamide**.**

### 4.2 Geographic distribution of resistant *Mycobacterium tuberculosis* isolates across western Kenya counties

**Figure 2:**
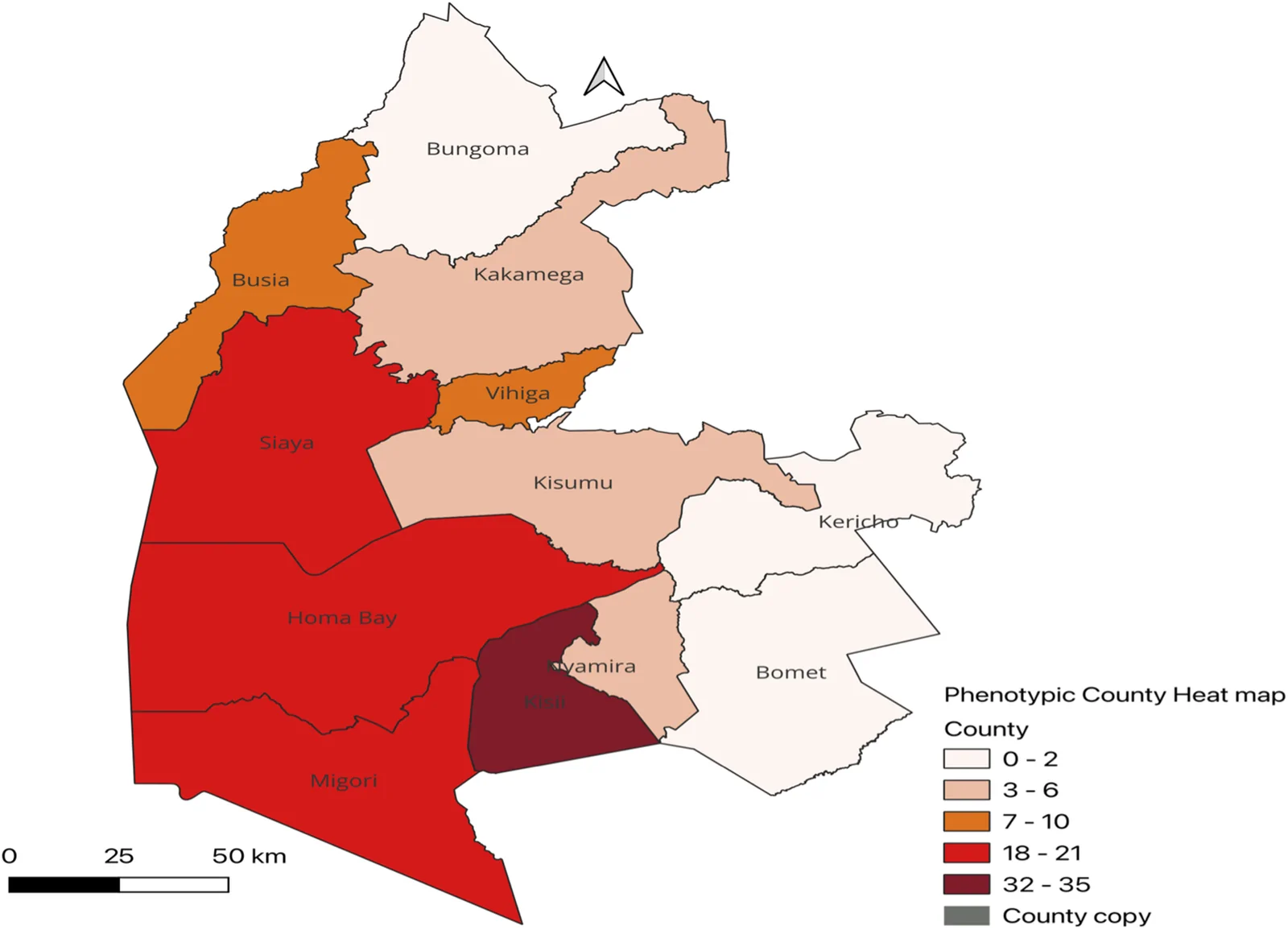
County-level heat map of line probe assay (LPA) and/or phenotypically drug-resistant *Mycobacterium tuberculosis* isolates across western Kenya (*n* = 127).

LPA and phenotypic DST results revealed significant geographic variation in drug resistance across western Kenya (Figure 2). Resistance was most concentrated in counties within the Lake Victoria basin, with the highest counts recorded in Kisii (*n* = 32), Homa Bay (*n* = 21), Migori (*n* = 20), and Siaya (*n* = 18).

Moderate numbers of resistant isolates were observed in Vihiga County (*n* = 10) and Busia County (*n* = 7). Lower counts of resistant isolates were identified in Kisumu (*n* = 6), Kakamega (*n* = 5), and Nyamira (*n* = 4). The lowest numbers of resistant isolates were recorded in Bungoma (*n* = 2) and Kericho (*n* = 2), while no resistant isolates were identified in Bomet County (*n* = 0). Overall, the majority of drug-resistant strains were concentrated within counties situated in the Lake Victoria basin.

### 4.3 Characteristics of the study isolates

Among the 290 isolates analyzed, the majority were from male patients (72%), and the median age was 34 years (IQR 26, 43). The largest proportion of isolates (58%) were from Individuals aged between 25 and 44. 50% of the isolates were from HIV negative patients while 36% were from people living with HIV (Table 2).

**Table 2:** Characteristics of the study isolates subjected to whole genome sequence analysis.

Characteristic**s**	*N* = 290 (%)
Age (years) (IQR)	34 (26–43)
Age category	
0–14	19 (6.6)
15–24	39 (13.0)
25–44	168 (58.0)
45–64	49 (17.0)
65+	15 (5.2)
Gender	
Female	80 (28.0)
Male	210 (72.0)
HIV status	
Positive	104 (36.0)
Negative	145 (50.0)
Declined/not done	41 (14.0)
County	
Bomet	6 (2.1)
Bungoma	6 (2.1)
Busia	9 (3.1)
Homa Bay	56 (19.0)
Kakamega	7 (2.4)
Kericho	2 (0.7)
Kisii	90 (31.0)
Kisumu	13 (4.5)
Migori	31 (11.0)
Nyamira	6 (2.1)
Siaya	32 (11.0)
Vihiga	32 (11.0)

Of 290 isolates, Kisii County contributed the largest proportion (*n* = 90, 31%)**,** followed by Homa Bay (*n* = 56, 19%) with moderate contributions from Siaya (*n* = 32, 11%), Vihiga (*n* = 32, 11%)**,** and Migori (*n* = 31, 11%)**.** The remaining counties each contributed <5% of isolates, including Kisumu (*n* = 13, 4.5%)**,** Busia (*n* = 9, 3.1%)**,** Bomet, Bungoma, and Nyamira (*n* = 6 each, 2.1%)**,** Kakamega (*n* = 7, 2.4%), and Kericho (*n* = 2, 0.7%) (Table 2)**.**

A total of 290 isolates were characterized using routine diagnostic methods available at the time of archiving. To validate the use of these mixed reference standards (LPA and pDST), a concordance analysis was performed on the subset of 121 isolates for which both results were available.

As shown in Table 3, there was a high degree of correlation between molecular and phenotypic methods, with an Overall Percent Agreement (OPA) of 84.3%. While the specificity of LPA was high (93.8%), the sensitivity compared to pDST was 65.9%, with 14 isolates identified as phenotypically resistant but molecularly sensitive by LPA.

**Table 3:** Concordance of drug susceptibility profiles between line probe assay (LPA) and phenotypic DST (pDST) for 121 MTBC isolates.

	LPA resistant	LPA sensitive	Total
Phenotypic resistant	27	14	41
Phenotypic sensitive	5	75	80
Total	32	89	121

These findings justify the inclusion of both LPA and pDST as routine reference data, while highlighting the diagnostic gaps that the subsequent Whole Genome Sequencing (WGS) analysis was utilized to resolve.

### 4.4 Geographic distribution of WGS-detected drug resistance among MTBC isolates

Among the 290 sequenced isolates, WGS identified resistance-associated mutations in 43 cases (14.8%). The demographic profile of these resistant cases was predominantly male (63%), HIV-positive (60%), and within the 25–44 age range (56%).

The geographic distribution of WGS-detected drug resistance varied across the study counties. Kisii County contributed the highest number of resistant isolates (12 of 43; 27.9%), followed by Migori County (8 isolates; 18.6%) and Homa Bay County (6 isolates; 14.0%). Additional resistant isolates were identified in Siaya County (5 isolates; 11.6%) and Busia County (4 isolates; 9.3%).

Lower numbers of resistant isolates were observed in Nyamira (3 isolates; 7.0%) and Kisumu (2 isolates; 4.7%). Single resistant isolates were detected in Kakamega (1), Vihiga (1), and Kericho (1), each accounting for 2.3% of the resistant isolates. In contrast, no WGS-detected drug resistance was observed among the isolates obtained from Bomet and Bungoma counties (Table 4).

**Table 4:** County-level distribution of WGS-detected drug-resistant *Mycobacterium tuberculosis* isolates in western Kenya.

No	WGS resistance distribution in Western Kenya		
	Counties	No. isolates(*n* = 290)	WGS resistance (%), *n* = 43
1	Kisumu	13	2 (4.7)
2	Migori	31	8 (18.6)
3	Siaya	32	5 (11.6)
4	Homa Bay	56	6 (14.0)
5	Kisii	90	12 (27.9)
6	Nyamira	6	3 (7.0)
7	Busia	9	4 (9.3)
8	Kakamega	7	1 (2.3)
9	Vihiga	32	1 (2.3)
10	Bomet	6	0 (0.0)
11	Bungoma	6	0 (0.0)
12	Kericho	2	1 (2.3)

### 4.5 Spectrum of resistance-conferring mutations by drug class

**Figure 3:**
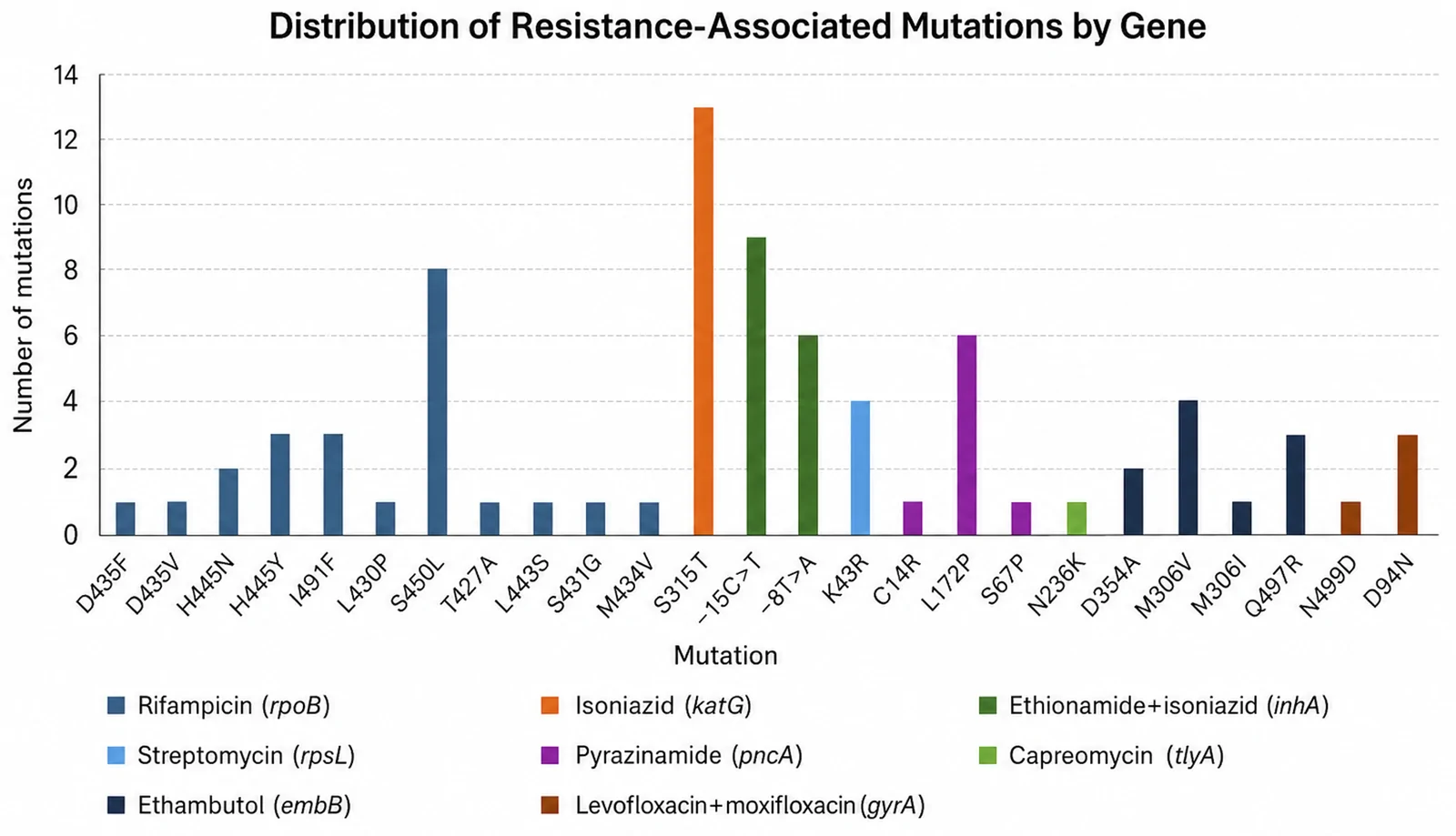
Distribution of resistance-associated gene mutations identified by whole-genome sequencing among drug-resistant *Mycobacterium tuberculosis* isolates from western Kenya.

Whole genome sequencing revealed a diverse spectrum of mutations across first- and second-line anti-tuberculosis drug resistance genes (Figure 3). For first-line agents, rifampicin and isoniazid resistance was predominantly mediated by key variants in *rpoB* (most commonly S450L) and *katG (*dominated by S315 T), respectively. Ethionamide resistance was observed through *inhA* promoter mutations (specifically −15 C > T and −8 T > A), which also contributed to isoniazid resistance. Pyrazinamide resistance was largely attributable to *pncA* mutations, primarily L172P, while *embB* mutations, particularly M306 V, accounted for most of the ethambutol resistance. Streptomycin resistance was associated with *rpsL* K43R. Second-line drug resistance was less frequent, mainly involving *gyrA* mutations linked to fluoroquinolone resistance, alongside a single *tlyA* mutation associated with capreomycin resistance. Notably, no resistance-conferring mutations were identified for newer or repurposed agents, including bedaquiline and clofazimine (*Rv0678*, *mmpL5*, *pepQ*) or cycloserine (*ald*, *alr*). Furthermore, all isolates were genotypically susceptible to the second-line injectables amikacin and kanamycin, with no mutations detected in the *rrs* gene or *eis* promoter (Figure 3). A comprehensive dataset containing the raw mutational analysis for all 43 isolates is provided in Supplementary Table 2.

### 4.6 Lineage and Sub-lineage distribution of drug-resistant isolates

Drug-resistant isolates were distributed across multiple MTBC lineages circulating in western Kenya. Lineage 4 accounted for the largest proportion of resistant isolates (22/121; 18.2%), followed by Lineage 3 (15/109; 13.8%), while Lineage 2 showed the lowest proportion (5/58; 8.6%); however, these differences were not statistically significant (*χ*^2^ = 2.19, *p* = 0.33). At the sub-lineage level, the highest absolute number of resistant isolates occurred in Lineage 3 group (10/43), followed by sub-lineage's 4.3.3 (8/43), 3.1.1 (5/43), and 2.2.1 (5/43). When considered relative to the number of isolates within each sub-lineage, resistance appeared particularly prominent in sub-lineages 4.3.3 and 4.6.1.2, although interpretation of several sub-lineages should be cautious due to very small sample sizes (Table 5).

**Table 5:** Distribution of WGS-detected drug-resistant *Mycobacterium tuberculosis* isolates by sub-lineage in western Kenya.

Sub lineage	Resistance count (%)	Total sub-lineage
Lineage 1.1.2	1 (100)	1
Lineage 2.2.1	5 (8.6)	58
Lineage 3	10 (23.3)	43
Lineage 3.1.1	5 (8.8)	57
Lineage 4.1.1.3	1 (100)	1
Lineage 4.1.2.1	2 (28.6)	7
Lineage 4.3.3	8 (80)	10
Lineage 4.3.4	1 (100)	1
Lineage 4.3.4.2.1	1 (11.1)	9
Lineage 4.4.1.1	3 (10.3)	29
Lineage 4.6	1 (33.3)	3
Lineage 4.6.1.2	4 (40)	10
Lineage 4.6.2	1 (100)	1
Total	43	230

Sub-lineages with *n* < 5 are shown for completeness, but percentage values should not be interpreted as stable estimates.

The 247 susceptible MTBC isolates were distributed across three major global lineages and the *M. bovis* species. The population was primarily dominated by Lineage 4 (*n* = 99; 40.1%) and Lineage 3 (*n* = 94; 38.1%). Lineage 2 accounted for the remaining significant portion of the susceptible cohort (*n* = 53; 21.5%), while a single isolate was identified as *M. bovis* (*n* = 1). This distribution highlights a diverse susceptible background in Western Kenya, where multiple co-circulating lineages act as the primary reservoir for MTBC transmission.

At the sub-lineage level, the susceptible isolates exhibited genetic granularity across several well-defined clades. The most prevalent sub-lineages identified were 3.1.1 (*n* = 43) and 2.2.1 (*n* = 43), followed by 4.4.1.1 (*n* = 17) and 4.3.4.2 (*n* = 14). Additionally, a substantial group was identified only at the broad Lineage 3 level (*n* = 31). Notably, all four mixed infections (*n* = 4; 1.6%) identified in the study occurred within this susceptible group, featuring complex combinations of distinct lineages, such as sub-lineage 4.2.2 with Lineage 3 and sub-lineage 2.2.1 with 1.1.2. For frequency and resistance analysis, these isolates were categorized according to their dominant strain as called by the TBProfiler LineageID.

### 4.7 Geographic distribution of drug-resistant MTBC sub-lineages in western Kenya

Drug-resistant isolates were distributed across several MTBC sub-lineages and counties in Western Kenya. Kisii County contributed the largest number of resistant isolates (*n* = 12), predominantly belonging to sub-lineage 4.3.3 (*n* = 6) and 4.4.1.1 (*n* = 3). Migori contributed eight resistant isolates mainly from Lineage 3 and sub-lineage 3.1.1, while Homa Bay exhibited resistance across multiple sub-lineages including sub-lineage 2.2.1 and sub-lineage 4.6.1.2. Other counties such as Busia, Siaya, Nyamira and Kisumu showed smaller numbers of resistant isolates distributed across different sub-lineages, whereas only single resistant isolates were detected in Vihiga, Kakamega, and Kericho (Supplementary Table 3).

### 4.8 WGS drug resistance profile

WGS identified resistance-associated mutations in 43 of the 290 sequenced MTBC isolates. Hr-TB represented the most frequent resistant isolates (16; 37%), followed by RR-TB (11; 25.6%) and MDR-TB (9; 20.9%). Three isolates were classified as pre-XDR-TB based on WGS-derived detection of fluoroquinolone resistance-associated mutations in MDR-TB isolates. This differed slightly from the routine diagnostic results presented in Table 2, where two pre-XDR-TB isolates were identified using LPA and/or phenotypic DST. Monoresistance to streptomycin, capreomycin, and fluoroquinolones was detected in a small number of isolates. The sequencing dataset was enriched for resistant isolates; therefore, these proportions represent the distribution of resistance phenotypes among the sequenced isolates rather than population-level prevalence (Table 6).

**Table 6:** Distribution of drug susceptibility detected by whole genome sequenced *Mycobacterium tuberculosis* isolates (*n* = 290) from western Kenya.

Drug resistance type	No. of isolates, *n* = 290 (%)
Drug-susceptible	247; 85.1%
Hr-TB	16 (5.5)
RR-TB	11 (3.8)
MDR-TB	9 (3.1)
pre-XDR-TB	3 (1.0)
STR-R	2 (0.7
CAP-R	1 (0.3)
FQ-R	1 (0.3)

Percentages are calculated relative to the total number of sequenced isolates (*n* = 290). The sequencing dataset was enriched for resistant isolates during sample selection; therefore, these proportions should not be interpreted as population-level prevalence estimates of drug resistance. Hr-TB, resistance to isoniazid with susceptibility to rifampicin; RR-TB, Rifampicin-resistant tuberculosis; MDR-TB, resistance to both rifampicin and isoniazid; pre-XDR-TB, indicating MDR-TB with additional resistance to fluoroquinolones; STR-R, Streptomycin resistant; CAP-R, Capreomycin-resistant and FQ-R, (Fluoroquinolone-resistant- non-MDR/RR).

### 4.9 Comparison of LPA and/or phenotypic DST and WGS-based resistance detection

LPA and/or phenotypic DST testing identified 56 resistant isolates, compared with 43 resistant isolates detected by WGS. Concordant resistance between phenotypic testing and WGS was observed in 35 isolates, while 21 isolates were phenotypically resistant but WGS sensitive, and 8 isolates were resistant by WGS but phenotypically sensitive.

A statistically significant difference was observed between the two methods (McNemar's *χ*^2^ with continuity correction = 4.97, *df* = 1, ***p*** = 0.026). Overall, routine resistance testing (LPA and/or phenotypic DST) identified a greater number of resistant isolates compared with WGS (Table 7).

**Table 7:** Comparison of WGS-predicted resistance and LPA and/or phenotypic DST among MTBC isolates (*n* = 290).

Method/Result	WGS resistant	WGS sensitive	Total
LPA and/or phenotypic resistant	35	21	56
LPA and/or phenotypic sensitive	8	226	234
Total	43	247	290

McNemar's chi-squared test with continuity correction, *χ*^2^ = 4.97, *df* = 1, *p* = 0.026.

### 4.10. Discordant resistance detection between LPA and/or phenotypic testing and whole-genome sequencing

Notable discordances in resistance detection were observed between routine testing (LPA **and/or** phenotypic DST) and WGS. Routine testing identified resistance in 21 isolates that WGS classified as susceptible, primarily for isoniazid (*n* = 8), rifampicin (*n* = 5), and pyrazinamide (*n* = 4). Genomic re-evaluation via the WHO catalogue revealed that 18 of these 21 discordant cases harbored mutations of uncertain significance, which likely explains the lack of WGS-based resistance calls.

Conversely, WGS identified resistance mutations [WHO catalogue v2 (35)] in 8 isolates classified as susceptible or not tested by routine methods. Of these, 6 were truly discordant (WGS-resistant but routine-susceptible), involving rifampicin (*n* = 3), isoniazid, fluoroquinolones, and capreomycin. Two additional isolates carried streptomycin resistance mutations that had not been evaluated by routine testing (Table 8).

**Table 8:** Discordant resistance detection between LPA and/or phenotypic DST and WGS.

Drug resistance type	Detection	
	LPA and/or phenotypic DST (*n* = 21)	WGS (*n* = 8)
Hr-TB (INH-resistant, RIF-susceptible TB)	8	1
RR-TB (RIF-resistant TB)	5	3
Ethambutol (EMB)	2	0
Pyrazinamide (PZA)	4	0
KAN/AMK/CAP/VIO	2	1(CAP)
Streptomycin (STR)	-	2
FQ-R (fluoroquinolone-resistant- non-MDR/RR)	0	1

Hr-TB, resistance to isoniazid with susceptibility to rifampicin; RR-TB, rifampicin-resistant tuberculosis MDR-TB, resistance to both rifampicin and isoniazid; pre-XDR-TB, indicating MDR, TB with additional resistance to fluoroquinolones; ETH, ethionamide resistant; PZA, pyrazinamide resistant; STR-R, Streptomycin resistant; CAP-R, capreomycin-resistant; FQ-R, fluoroquinolone-resistant- non-MDR/RR; KAN, kanamycin; AMK, amikacin; CAP, capreomycin; VIO, viomycin.

## 5 Discussion

Drug-resistant tuberculosis remains a critical public health challenge in Kenya and across sub-Saharan Africa. The primary take-home messages of this study are twofold: first, there is a high predominance of Hr-TB and a heavy concentration of drug-resistant MTBC isolates within the Lake Victoria basin of Western Kenya; second, there is a significant discordance between WGS and routine diagnostic testing, largely driven by mutations currently classified by the WHO as having uncertain significance**.** While WGS successfully captured many canonical resistance-conferring mutations, these unclassified variants present a challenge for genomic surveillance. Furthermore, our findings confirm that drug-resistant TB is heterogeneously distributed across the region, heavily concentrated in specific counties.

While the 4th National Anti-tuberculosis DR-TB survey and other regional studies frequently attribute resistance to mutations in *katG*, *inhA*, and *rpoB*, Hr-TB remains an under-recognized challenge globally estimated at 7.4%–15.7% of cases (36, 37). This high prevalence suggests that rifampicin-based diagnostics, like Xpert MTB/RIF, miss a substantial portion of isoniazid resistance (38, 39). Conversely, some studies in Kenya and Uganda report higher proportions of MDR-TB than Hr-TB (10, 18, 40). This discrepancy likely reflects surveillance bias, as higher MDR-TB proportions are typically reported in referral cohorts and treatment failures rather than routine surveillance populations. The resistance profiles in this study align with established TB biology (41). Rifampicin resistance was primarily driven by *rpoB* S450L, while isoniazid resistance was dominated by the canonical *katG* S315 T and *inhA* promoter mutations. These findings reinforce these loci as the most reliable markers for first-line resistance prediction (12, 25, 41, 42). Second-line resistance markers, including *gyrA* and *tlyA*, also matched established mechanisms (41).

Resistant isolates were concentrated in the Lake Victoria basin (Kisii, Migori, Homa Bay, and Siaya), suggesting localized transmission or shared epidemiologic drivers (18, 43). The predominance of Lineage 4 and Lineage 3 is consistent with their known distributions in Africa and East Africa, respectively. While no significant association between lineage and resistance was found, the observed diversity suggests complex transmission dynamics and indicates that drug resistance is occurring across diverse circulating MTBC populations rather than a single dominant strain (44, 45). Similar patterns reported across Kenya and East Africa emphasize the need for granular, local genomic surveillance over a reliance on national averages (5, 10, 25). The detection of *M. bovis* underscores the public health importance of zoonotic TB surveillance, as transmission may occur through infected cattle or unpasteurized dairy products (46).

A significant discordance was observed, with routine methods detecting more resistant isolates than WGS. This gap is largely attributed to mutations currently classified by the WHO as having uncertain significance (41, 42). Such discrepancies are common for drugs like ethambutol and pyrazinamide, where resistance mechanisms are less defined (25, 47, 48). Factors like borderline resistance, heteroresistance, or uncurated mutations may also contribute (28). Ultimately, genomic and phenotypic methods play complementary roles, with WGS providing high-resolution mutation detection while phenotypic DST remains essential for confirming resistance in specific cases (12, 25, 47).

The observed discordance between genotypic predictions and phenotypic drug susceptibility testing (pDST) in this study also highlights the multifaceted nature of *Mycobacterium tuberculosis* resistance. Methodologically, the reliance on culture-dependent WGS may introduce selection bias, where *in vitro* conditions favor specific subpopulations that do not fully represent the heteroresistance present in the clinical isolate (49). Biologically, current genomic models cannot account for non-genetic mechanisms such as transient metabolic shifts, increased efflux pump activity, or reduced cell wall permeability (50, 51). Furthermore, the clinical interpretation is hampered by the lack of global standardization for Phenotypic Drug Susceptibility Testing (pDST) regarding ethionamide (ETH), pyrazinamide (PZA), and fluoroquinolones (FQ), where technical variables like pH sensitivity and narrow critical concentrations frequently lead to inconsistent phenotypic results (52). These factors emphasize that while WGS is a transformative diagnostic tool, it must be interpreted alongside an understanding of these inherent biological and technical limitations.

## 6 Limitations of the study

This study is subject to several limitations. The retrospective design relied on archived referral laboratory isolates, potentially introducing a selection bias toward drug-resistant cases rather than population prevalence. Furthermore, the cross-sectional nature of the research precludes direct inferences regarding transmission dynamics. Notably, the analysis was constrained by the lack of SNP-based linkage analysis; while lineage and sub-lineage distributions were identified, the absence of genomic clustering based on single nucleotide polymorphism (SNP) thresholds limits the ability to definitively identify recent transmission chains or localized outbreaks. The analysis was also constrained by the absence of routine testing for specific drugs like streptomycin and a lack of clinical outcome data, which coupled with the evolving nature of the WHO mutation catalogue limits the assessment of prognostic implications for variants of uncertain significance.

## 7 Conclusion

This study offers critical insights into the molecular epidemiology of drug-resistant *M. tuberculosis* in western Kenya, identifying isoniazid-resistant, rifampicin-susceptible (Hr-TB) as the most prevalent resistance pattern. Using WGS, key mutations were identified in genes such as *rpoB, katG, inhA, pncA, embB,* and *gyrA*, with resistant isolates showing broad distribution across lineages and counties, particularly within the Lake Victoria basin. A significant discordance between routine testing and WGS was noted, primarily attributed to variants of uncertain significance in the WHO mutation catalogue. These results emphasize that rifampicin-biased diagnostic tools likely under-detect a substantial number of Hr-TB cases and suggest that the clinical utility of WGS is currently limited by incomplete global genomic databases.

## 8 Recommendations

Integration of whole-genome sequencing into national TB surveillance systems could strengthen detection and monitoring of drug-resistant TB in Kenya. In the short term, this should prioritize the implementation of WGS at central reference laboratories while simultaneously improving the detection of isoniazid resistance, given the high prominence of Hr-TB identified in this study. Furthermore, WGS and phenotypic DST should continue to be used in a complementary manner, particularly for drugs with less well-defined resistance mechanisms. Finally, continued generation and integration of local genomic and phenotypic data will be essential for refining global resistance mutation catalogues and improving genomic resistance prediction in high-burden settings.

## Data Availability

The Whole-Genome Sequencing (WGS) datasets generated for the 290 Mycobacterium tuberculosis complex isolates in this study are publicly available in the National Center for Biotechnology Information (NCBI) BioProject database under accession number PRJNA1462751 (https://www.ncbi.nlm.nih.gov/bioproject/PRJNA1462751).
