# Supplementary material for: Genomic characterization of drug-resistant Mycobacterium tuberculosis complex in western Kenya reveals predominance of isoniazid resistance and discordance with routine susceptibility testing
Source: Front Tuberc. 2026 Jun 11;4:1855780. doi: 10.3389/ftubr.2026.1855780 (PMC13491443; doi:10.3389/ftubr.2026.1855780)
Supplement: Supplementary file 1 [file Table1.docx]

**Supplementary Table 1: Isolate Distribution by County, Year, and Study Dataset**

|  | County | 2021 | | 2022 | | Total Collection | Total Sequenced |
| --- | --- | --- | --- | --- | --- | --- | --- |
|  |  | **Total Collection** | **Sequenced Subset** | **Total Collection** | **Sequenced Subset** |  |  |
| 1 | **Bomet** | 0 | 0 | 21 | 6 | 21 | 6 |
| 2 | **Bungoma** | 1 | 0 | 17 | 6 | 18 | 6 |
| 3 | **Busia** | 17 | 7 | 20 | 2 | 37 | 9 |
| 4 | **Homa Bay** | 84 | 18 | 127 | 38 | 211 | 56 |
| 5 | **Kakamega** | 6 | 0 | 20 | 7 | 26 | 7 |
| 6 | **Kericho** | 2 | 0 | 6 | 2 | 8 | 2 |
| 7 | **Kisii** | 157 | 53 | 168 | 37 | 325 | 90 |
| 8 | **Kisumu** | 14 | 5 | 25 | 8 | 39 | 13 |
| 9 | **Migori** | 64 | 17 | 55 | 14 | 119 | 31 |
| 10 | **Nyamira** | 8 | 3 | 10 | 3 | 18 | 6 |
| 11 | **Siaya** | 59 | 14 | 54 | 18 | 113 | 32 |
| 12 | **Vihiga** | 31 | 9 | 87 | 23 | 118 | 32 |
|  | **Total** | **443** | **126** | **610** | **164** | **1,053** | **290** |
