# Supplementary material for: Genomic characterization of drug-resistant Mycobacterium tuberculosis complex in western Kenya reveals predominance of isoniazid resistance and discordance with routine susceptibility testing
Source: Front Tuberc. 2026 Jun 11;4:1855780. doi: 10.3389/ftubr.2026.1855780 (PMC13491443; doi:10.3389/ftubr.2026.1855780)
Supplement: Supplementary file 2 [file Table3.docx]

**Supplementary Table 3. County-level distribution of WGS-detected drug-resistant *Mycobacterium tuberculosis* sub-lineages in Western Kenya (n = 43).**

| **County** | **L1.1.2** | **L2.2.1** | **L3** | **L3.1.1** | **L4.1.2.1** | **L4.1.1.3** | **L4.3.3** | **L4.3.4** | **L4.3.4.2.1** | **L4.4.1.1** | **L4.6** | **L4.6.1.2** | **L4.6.2** | **Total** |
| --- | --- | --- | --- | --- | --- | --- | --- | --- | --- | --- | --- | --- | --- | --- |
| **Kisii** | **0** | **0** | **1** | **1** | **1** | **0** | **6** | **0** | **0** | **3** | **0** | **0** | **0** | **12** |
| **Migori** | **0** | **2** | **4** | **2** | **0** | **0** | **0** | **0** | **0** | **0** | **0** | **0** | **0** | **8** |
| **Homa Bay** | **0** | **1** | **0** | **0** | **0** | **0** | **0** | **0** | **1** | **0** | **1** | **3** | **0** | **6** |
| **Busia** | **1** | **1** | **1** | **0** | **1** | **0** | **0** | **0** | **0** | **0** | **0** | **0** | **0** | **4** |
| **Siaya** | **0** | **1** | **2** | **0** | **0** | **0** | **2** | **0** | **0** | **0** | **0** | **0** | **0** | **5** |
| **Nyamira** | **0** | **0** | **1** | **0** | **0** | **0** | **0** | **1** | **0** | **0** | **0** | **0** | **1** | **3** |
| **Kisumu** | **0** | **0** | **1** | **1** | **0** | **0** | **0** | **0** | **0** | **0** | **0** | **0** | **0** | **2** |
| **Vihiga** | **0** | **0** | **0** | **0** | **0** | **1** | **0** | **0** | **0** | **0** | **0** | **0** | **0** | **1** |
| **Kakamega** | **0** | **0** | **0** | **1** | **0** | **0** | **0** | **0** | **0** | **0** | **0** | **0** | **0** | **1** |
| **Kericho** | **0** | **0** | **0** | **0** | **0** | **0** | **0** | **0** | **0** | **0** | **0** | **1** | **0** | **1** |
